# Travis County Medical Society Proceedings

**Published:** 1889-12

**Authors:** 


### TRAVIS COUNTY MEDICAL SOCIETY—STATED MEETING, OCTOBER 3, 1889.

The President, Dr. John Preston, in the chair. Dr. J. F. Dean, of Hornsby, reported several cases of mastitis treated by electricity. He applied the Faradic current directly to the gland by means of sponge electrodes, in the beginning; if there is much pain and swelling, two or three seances, of fifteen or twenty minutes each, are given in twenty-four hours, and claims that this line of treatment, if begun early, will prevent suppuration.

In all of these cases—a short time after the application of the electricity—a free flow of milk was produced, with almost immediate relief of the pain.

The main subject for the evening: "Malaria and its Treatment," was next called up. In the absence of a paper, Dr. J. W. McLaughlin was invited to make some remarks on the subject. He reviewed, in a very thorough manner, the pathology and various theories as to the causation of the disease, and gave support to the microbian theory. For treatment, thought some form of mercury and quinine gave the best results.

Dr. Bennett thought the disease a neurosis, and opposed the microbian theory. Periodicity can be seen everywhere in nature; and could not understand how this characteristic feature of malarial toxæmia could prove the existence of a specific cause. He had seen results from salicylate of soda and belladonna in cases where, from some idiosyncracy, quinine could not be used. Ipecacuanha is a good remedy.

Dr. Q. C. Smith remarked that the exacerbation of symptoms at regular intervals was a peculiar characteristic of nearly all diseases which affect the liver. In the treatment of malarial fevers thought it unnecessary to give large doses of quinine; that more pleasant and better results could be obtained from small doses— not more than two or three grains—with other remedies which are known to have an effect upon this disease, as gelseminum, hydrastis, ipecacuanha, etc.

Dr. Litten didn't agree with Dr. Smith in the use of small doses of quinine, as much is gained by promptly controlling the paroxysm; he thought, ordinarily, twenty grains before a paroxysm was the proper dose.

Dr. T. O. Maxwell also favored large doses of quinine—fifteen or twenty grains three hours before the time of a paroxysm. Not safe to give pregnant women that amount. Was confident he had seen abortion produced by it.

Dr. Wooten remarked that the growth of vegetation in malarial districts were salutary, as the poison was probably absorbed by them as it came from the ground, and either destroyed or rendered less virulent. For treatment he recommended twenty or thirty grains of quinine before the paroxysm. Not necessary to wait for the fever to subside before giving the quinine. He generally used massa hydrargyrum and rhubarb for a purgative.

It being the time for the annual election of officers, nominations were made, and the society proceeded to ballot, which resulted in the election of the following officers for the ensuing year:

Dr. T. J. Tyner, President; Dr. J. A. Lewright, Vice-President; Dr. B. F. Church, Secretary and Treasurer; Drs. J. W. McLaughlin, R. M. Swearingen and J. M. Litten, Censors.

It was unanimously agreed that the society meet every two weeks through the winter.

Two subjects were selected for the next meeting: "Mastitis and Salpingitis," and essayists appointed to present them. Adjourned to meet Oct. 17.

